# Basic science of osteoarthritis

**DOI:** 10.1186/s40634-016-0060-6

**Published:** 2016-09-13

**Authors:** Magali Cucchiarini, Laura de Girolamo, Giuseppe Filardo, J. Miguel Oliveira, Patrick Orth, Dietrich Pape, Pascal Reboul

**Affiliations:** 1 Center of Experimental Orthopaedics, Saarland University Medical Center and Saarland University, Kirrbergerstr. Bldg 37, D-66421 Homburg, Germany; 2 Orthopaedic Biotechnology Laboratory, Galeazzi Orthopaedic Institute, Milan, Italy; 3 Orthopaedic and Traumatologic I Clinic, Biomechanics Laboratory, Rizzoli Orthopaedic Institute, University of Bologna, Bologna, Italy; 4 3B’s Research Group - Biomaterials, Biodegradables and Biomimetics, Univ. Minho, Headquarters of the European Institute of Excellence on Tissue Engineering and Regenerative Medicine, Avepark - Parque de Ciência e Tecnologia, Zona Industrial da Gandra, Barco GMR, Barco, Guimarães, Portugal; 5 ICVS/3B’s - PT Government Associated Laboratory, Barco, Guimarães, Portugal; 6 Department of Orthopaedic Surgery, Saarland University Medical Center and Saarland University, Homburg, Saar Germany; 7 Department of Orthopaedic Surgery, Centre Hospitalier de Luxembourg, Luxembourg ville, Luxembourg; 8 Sports Medicine Research Laboratory, Public Research Centre for Health, Luxembourg, Centre Médical de la Fondation Norbert Metz, Luxembourg ville, Luxembourg; 9 UMR 7365 CNRS-Université de Lorraine, IMoPA, Biopôle de l’Université de Lorraine, Campus Biologie-Santé, Vandoeuvre-lès-Nancy, France

**Keywords:** Osteoarthritis, Articular cartilage, Bone, Animal models, Pathomechanisms, Interface, Stem cells, Tissue engineering

## Abstract

Osteoarthritis (OA) is a prevalent, disabling disorder of the joints that affects a large population worldwide and for which there is no definitive cure. This review provides critical insights into the basic knowledge on OA that may lead to innovative end efficient new therapeutic regimens. While degradation of the articular cartilage is the hallmark of OA, with altered interactions between chondrocytes and compounds of the extracellular matrix, the subchondral bone has been also described as a key component of the disease, involving specific pathomechanisms controlling its initiation and progression. The identification of such events (and thus of possible targets for therapy) has been made possible by the availability of a number of animal models that aim at reproducing the human pathology, in particular large models of high tibial osteotomy (HTO). From a therapeutic point of view, mesenchymal stem cells (MSCs) represent a promising option for the treatment of OA and may be used concomitantly with functional substitutes integrating scaffolds and drugs/growth factors in tissue engineering setups. Altogether, these advances in the fundamental and experimental knowledge on OA may allow for the generation of improved, adapted therapeutic regimens to treat human OA.

## Introduction

**Fig. 1:**
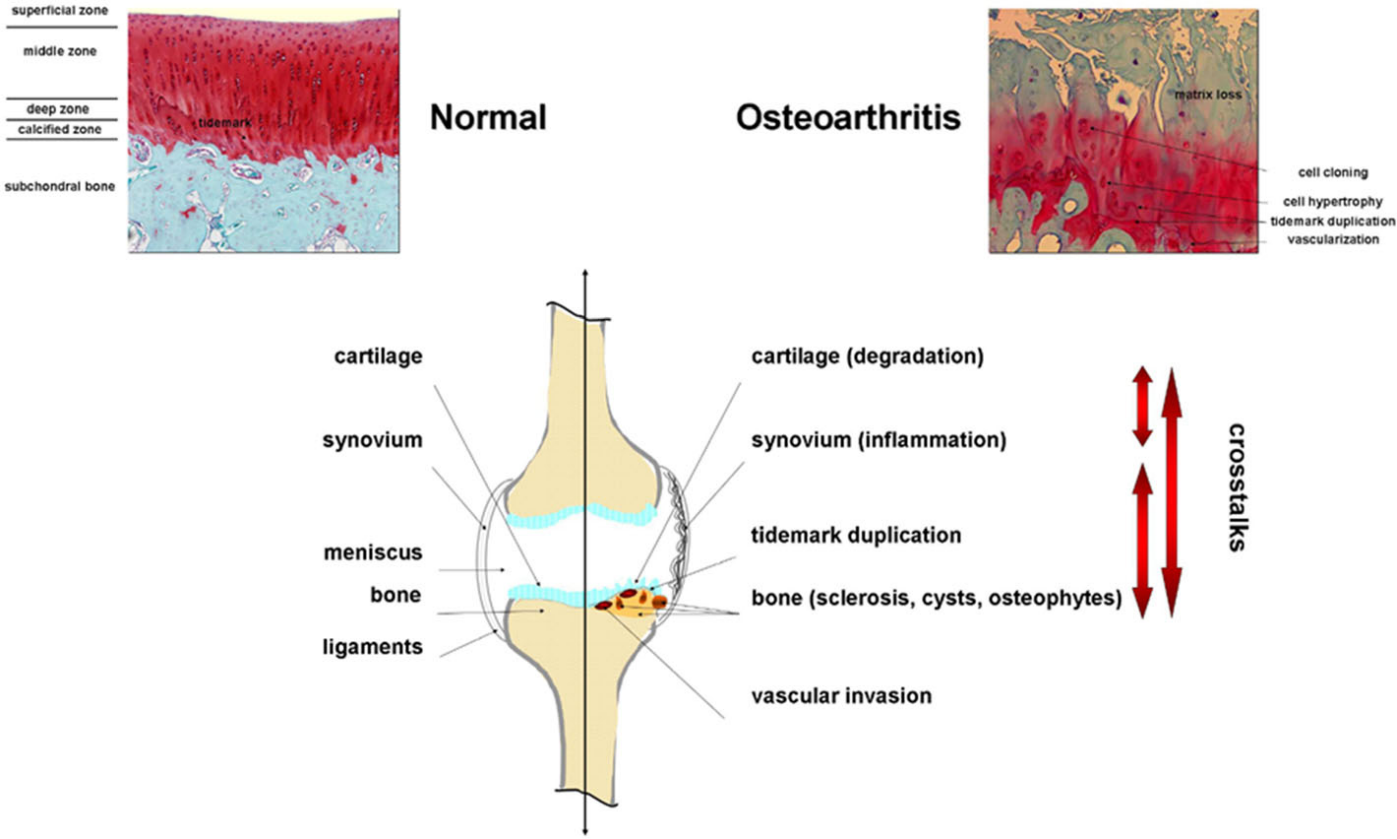
OA-associated changes in the human joint. Safranin O-stained histological sections from human normal versus OA knee joint cartilage are presented on the top panels. The lower panel is a representation of the different tissues implicated in the OA pathology with crosstalks

Osteoarthritis (OA) is a systemic, chronic joint disorder classified into primary and secondary OA depending on its etiology, characterized by the progressive breakdown of the articular cartilage (the end point of OA) along with changes in the subchondral bone, synovium (synovial inflammation), meniscus, tendons/ligaments, and muscles (Fig. 1) (Loeser et al. 2012). OA is a multifactorial disorder that can be divided in non-genetic (age, gender, obesity, mechanical stress, inactive lifestyle, joint trauma, patient occupational activities) and genetic (altered gene expression patterns of the cartilage and subchondral bone) factors. It can affect any joint of the body but most significantly weightbearing joints of lower extremities and the hand, causing pain and impaired functionality in the adult population.

OA is a common condition limiting the quality of life of patients, representing a social, economic burden (Bijlsma et al. 2011). Its prevalence increases with aging and obesity. Knee OA affects over 70 million Europeans, and the direct costs exceed 2 billion euros. The disease progression is commonly slow but ultimately leads to joint malfunction as the cartilage has a poor regeneration capability. According to World Health Organization (WHO) Global Burden of Disease Study 2010, hip and knee OA is the 11^th^ leading cause of disability.

Traditional treatments, apart from exercise and weight loss, include non-steroidal anti-inflammatory drugs (NSAIDs), supplements (glucosamine, chondroitin), viscosupplementation, arthroscopic lavage and debridement, combined non-pharmacological and pharmacological modalities, and replacement surgery as a sustainable alternative in restricted situations. Still, none of these procedures proved successful so far to fully reverse the OA phenotype in patients, mostly because the pathogenesis of the disease is still poorly understood.

On May 7^th^, 2016, clinicians and basic scientists from Europe, North America, and Asia met on the occasion of an Instructional Course Lecture (ICL16: *Basic science of osteoarthritis*) at the 17^th^ European Society of Sports Traumatology, Knee Surgery & Arthroscopy (ESSKA) Congress in Barcelona, Spain to explore the current knowledge of OA. The purpose of the present contribution is to highlight new, critical aspects of clinically relevant, scientific and translational OA research based on individual presentations from orthopaedic surgeons, cell and molecular biologists and biochemists, biomaterial scientists and engineers (Table 1) that may help define new pathways underlying OA pathogenesis and subsequently to identify novel, potential targets for OA therapy.

**Table 1:** Lectures of the ICL16 on May 7^th^, 2016 of the 17^th^ ESSKA Congress in Barcelona, Spain

Speaker	City	Country	Title of lectures
Magali Cucchiarini	Homburg/Saar	Germany	*Cell-matrix interface in OA*
Laura de Girolamo	Milano	Italy	*Animal models of OA*
Giuseppe Filardo	Bologna	Italy	*Stem cells in OA*
J. Miguel Oliveira	Guimarães	Portugal	*Tissue engineering in OA*
Patrick Orth	Homburg/Saar	Germany	*The bone in OA*
Dietrich Pape	Luxembourg	Luxembourg	*High tibial osteotomy models*
Pascal Reboul	Nancy	France	*OA pathomechanisms*

## The bone in OA

The chronological events controlling the pathology of OA are still debated but recent developments emphasized an early pathophysiological role of the subchondral bone. The subchondral bone, located immediately below the layer of calcified cartilage, forms the osteochondral unit together with the articular cartilage. The basophilic line on histological sections separating the hyaline cartilage from the underlying calcified cartilage is the tidemark while the border that separates the calcified cartilage from the subchondral bone plate is the cement line. Two parts constitute the subchondral bone. The subchondral bone plate is composed of a cortical bone plate and cancellous bone, which join together to enclose narrow intervening spaces. The highly mineralized cortical plate is made of osteon units containing a central Haversian canal with nerves and capillaries. Osteon units communicate with each other by Volkmann canals. The trabecular bone modelling medullar cavities instead have a level of mineralization of only about 15–25 % bone volume. The intervening spaces are gradually enlarged and become elongated in deeper regions of the subchondral bone, forming the subarticular spongiosa (Orth et al. 2013). Subchondral cortical and cancellous bone are architecturally, physiologically and mechanically different and respond differently in OA (Burr & Gallant 2012).

Radin and co-workers first identified the subchondral bone as a key factor in the initiation and progression of OA (Radin et al. 1970, Radin et al. 1972), showing that repetitive impulsive joint loading causes trabecular microfractures in the subchondral bone. Repair of such microfractures was thought to increase stiffness variation in the subchondral bone and in turn, cause tension and shear stress on the cartilage, ultimately leading to joint degeneration. However, several investigations revealed that the subchondral bone in OA is subjected to a decrease rather than an increase in bone stiffness (Burr 2004, Day et al. 2001). Besides, this strictly mechanical approach and the historical interpretation of the role of the subchondral bone can not explain the initial subchondral bone loss observed in early OA, do not allow for differentiation between early and late stages of the disease, and do neither address biological aspects nor the role of the calcified cartilage. Thus, a better in-depth understanding of the complex processes involved in OA is mandatory.

Current knowledge allows differentiation of two distinct pathophysiological processes: early and late stage OA. Early OA is clinically defined as the combination of joint pain, minor radiographic changes, but also presence of cartilage or subchondral bone lesions as seen by arthroscopy or magnetic resonance imaging (Luyten et al. 2012, Madry et al. 2016). Bone scintigraphic analyses (Buckland-Wright et al. 1991) or longitudinal studies following anterior cruciate ligament rupture (Buckland-Wright et al. 2000) suggest that subchondral bone changes may precede articular cartilage degeneration in OA initiation.

Remodelling processes play the crucial role in the pathogenesis of early OA and determine apparent subchondral bone changes (Burr 2004). Here, activation of secondary ossification centers, e.g. due to subchondral microfractures, results in bone resorption by osteoclasts followed by bone formation by osteoblasts. While this remodelling sequence is strictly balanced under physiological conditions, the rate of bone turnover is increased three- to fivefold in early OA (Amir et al. 1992). Importantly, in such high rates of bone turnover, the late phase of tissue mineralization is attenuated, leading to decreased bone stiffness (Goldring & Goldring 2010). Enhanced vascularization of the subchondral bone plate (Burr & Gallant 2012) causes decreased bone deposition and reduced its thickness (Intema et al. 2010). Such decreased thickness has been confirmed in canine and lapine experimental models of early OA (Bellido et al. 2011, Sniekers et al. 2008). Causes of increased bone remodelling include cellular signalling processes for microdamage repair (Funck-Brentano & Cohen-Solal 2011), stimulation of vascular invasion by angiogenic factors (Pesesse et al. 2011), and disturbed bone-cartilage crosstalk via physiological subchondral pores (Duncan et al. 1987) or pathological microcracks (Burr & Radin 2003).

The layer of calcified cartilage in normal joints is more dense than bone and thinner than the overlying hyaline articular cartilage (ratio 10:1) (Burr 2004). In early OA, the calcified cartilage layer is subjected to increased endochondral ossification (Suri & Walsh 2012), most likely triggered by vascular invasion from the subchondral bone compartment. In addition, recent experimental data suggest that hypertrophic chondrocytes within the calcified cartilage may transdifferentiate into osteoblasts, thus directly obtaining the capacity for bone formation (Yang et al. 2014, Zhou et al. 2014). Such ossification results in an accelerated advancement and duplication of the tidemark with increased thickness of the calcified cartilage layer. This process may increase the mechanical stress in the deep zones of the hyaline articular cartilage, contributing to the acceleration of OA (Goldring & Goldring 2010).

In late stage OA, the bone remodelling sequence is decreased and bone turnover is reduced. This allows for an increase in bone deposition, resulting in an enhanced thickness of the subchondral bone plate and an increased density of cortical and cancellous subchondral bone (Burr & Gallant 2012). This phenomenon is clinically known as subchondral sclerosis (Kellgren & Lawrence 1957). Interestingly, osteoblasts in late stage OA secrete a type-I collagen α1 homotrimer phenotypically distinct from the normal α1/α2 heterotrimer (Chan et al. 2011, Couchourel et al. 2009) and possessing a reduced affinity for calcium. This resolves the paradox of increased subchondral bone density relative to decreased bone mineralization and stiffness in late OA.

## OA pathomechanisms (focus on the subchondral bone)

Early-stage OA increases remodelling and bone loss of both trabecular and cortical subchondral entities (the two parts of the subchondral bone), whereas late-stage OA decreases remodelling and increases subchondral plate densification (Burr & Gallant 2012).

Trabecular and bone plate are composed of osteocytes, osteoblasts, and osteoclasts that function in a coordinated manner during bone homeostasis. However, these cells become malfunctioning during OA and most particularly the osteoblasts that acquire an OA phenotype especially in advanced OA. Such OA osteoblastic phenotype is more marked in the subchondral bone plate than in the trabecular bone, located at one centimeter of the cortical plate (Lavigne et al. 2005), and consisting in the up-regulation of numerous genes coding for extracellular matrix proteins, secreted cytokines and growth factors, and cell membrane proteins. For instance, interleukins 6 and 8 (IL-6, IL-8), leptin, and prostaglandins E_2_ (PGE_2_) are inflammatory factors secreted in a higher amount by OA osteoblasts (Hilal et al. 1998, Lisignoli et al. 2006, Massicotte et al. 2002, Mutabaruka et al. 2010). Type-I collagen (the major protein of the osteoid matrix) as well osteocalcin and osteopontin (proteins involved in matrix mineralization) are also produced in higher quantity (Gevers & Dequeker 1987, Hilal et al. 1998, Sanchez et al. 2008). However, OA bone is undermineralized (Grynpas et al. 1991), probably due to a defect of osteoblast function (Couchourel et al. 2009, Taylor et al. 2014). One explanation of this phenomenon could be that although type-I collagen production is increased (Mansell & Bailey 1998), the presence of homotrimers of alpha 1 chain of type-I collagen (Couchourel et al. 2009) instead of the classical 2-to-1 ratio of alpha 1 and alpha 2 chains disturbs the mineralization. Dickkopf-related protein 2 (DDK2) and sclerostin (SOST) whose expression increases, while that of R-spondin 2 (R-Spo2) decreases, seem also to play a role in the hypomineralization of OA bone (Abed et al. 2011, Abed et al. 2014, Chan et al. 2011). Interestingly, DKK1 is not differentially expressed between normal and OA osteoblasts (Chan et al. 2011). In addition to these factors, some cell membrane proteins involved in the balance of inorganic pyrophosphate and phosphate ions, namely alkaline phosphatase and ANKH (an inorganic pyrophosphate transport regulator) are upregulated during OA (Hilal et al. 1998, Sanchez et al. 2008). OA osteoblasts produce high level of growth factors such as transforming growth factor beta (TGF-β) (Mansell & Bailey 1998), insulin-like growth factor I (IGF-I) (Hilal et al. 1998, Massicotte et al. 2002), and hepatocyte growth factor (HGF) (Guevremont et al. 2003) as well as leptin (Mutabaruka et al. 2010) that may intervene in the modifications of the behavior of the articular chondrocyte through diffusion via microcracks. For example, TGF-β can either stimulate (Smad1/5/8 signaling pathway) or inhibit (Smad2/3 signaling pathway) chondrocyte terminal differentiation. There is further evidence that a switch in TGF-β signaling from Smad2/3 to Smad1/5/8 signaling plays a role in altered articular chondrocyte behavior and in the development of OA (van der Kraan et al. 2009). It was also shown that HGF can upregulate the expression of collagenase 3, a very active enzyme directed against type-II collagen (Reboul et al. 2001) whereas leptin is associated with OA cartilage (Dumond et al. 2003). Although the presence of these microcracks and of a greater number of blood vessels, OA subchondral bone undergoes hypoxic conditions. Very few studies have addressed hypoxia as measuring oxygen tension in the bone is arduous. However, venous stases (Arnoldi et al. 1972) have long been reported in OA patients and have been confirmed more recently with dynamic contrast-enhanced magnetic resonance imaging (MRI) (Aaron et al. 2007) and positron electron transfer (Dyke & Aaron 2010), sometimes leading to intra-osseous hypertension (Arnoldi et al. 1972). These perfusion abnormalities have also been associated with osteonecrosis and bone marrow lesions (Aaron et al. 2007) as dynamic episodes seen with MRI, often predicting progressive OA (Driban et al. 2013, Hunter et al. 2011, Pelletier et al. 2013, Xu et al. 2012).

Hypoxic conditions induce a high level of leptin production by osteoblast particularly under the control of vitamin D (Bouvard et al. 2014).

**Fig. 2:**
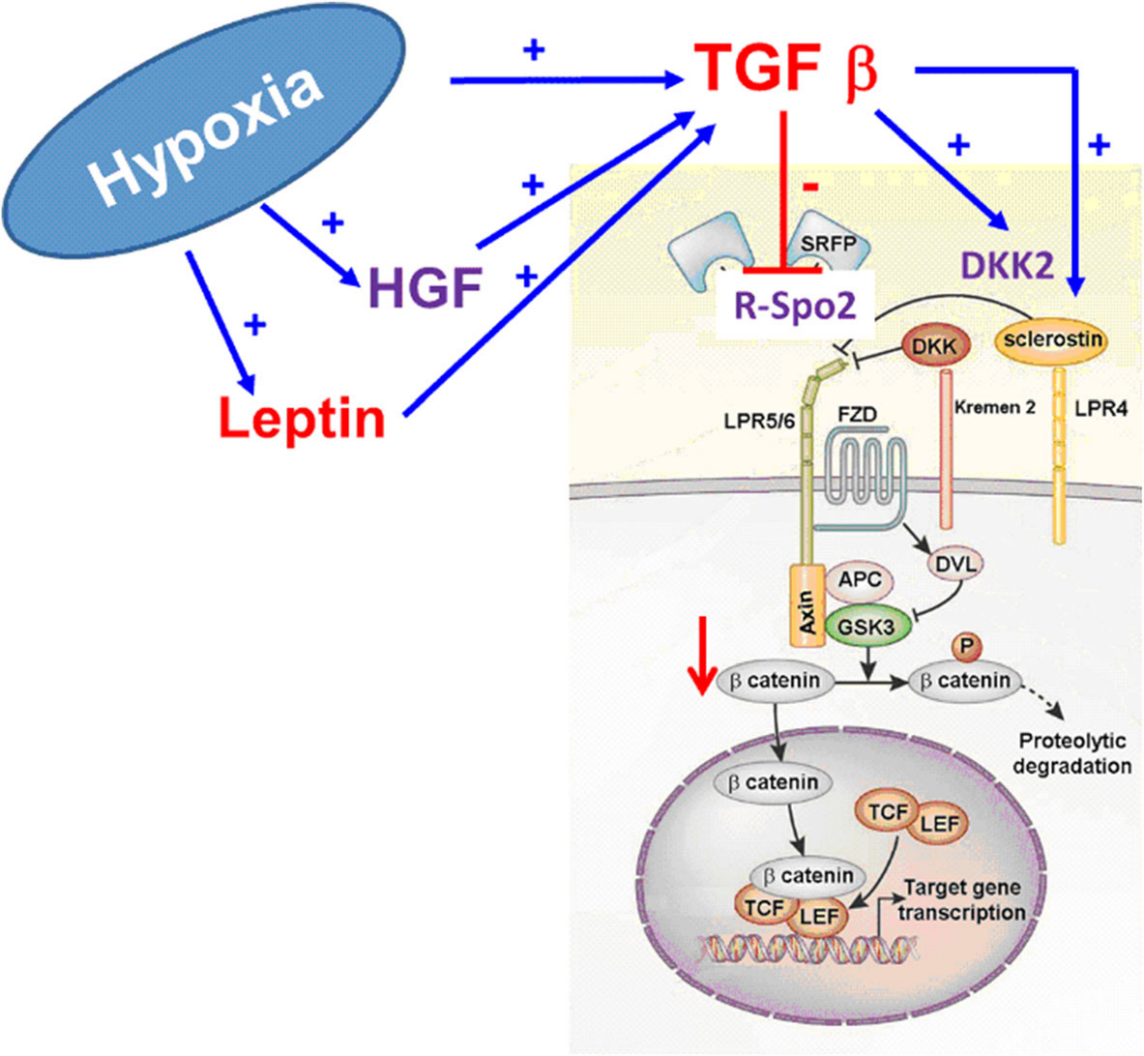
Factors regulating Wnt signaling in OA osteoblasts (modification from the original drawing representing the canonical Wnt route adapted from Baron & Kneissel 2013). APC, Adenomatous polyposis coli; DKK2, Dickkopf-related protein 2; DVL, Dishevelled protein; FZD, frizzled receptor; GSK3, glycogen synthase kinase 3; HGF, hepatocyte growth factor; LEF, lymphoid enhancing factor; LPR, lipoprotein receptor-related protein; R-Spo2, R-spondin 2; TCF, T-cell factor; TGF-β, transforming growth factor beta

Cell signaling in OA osteoblasts is disturbed too. For instance, OA osteoblasts show blunted cAMP synthesis in response to human parathyroid hormone and PGE_2_ in contrast to findings in normal cells, a result that is not attributed to altered adenylate cyclase activity (Hilal et al. 1998). Upon IGF-I stimulation, the phosphorylation of the IGF-I receptor (IGF-IR) is normal while that of the insulin receptor substrate 1 (IRS-1) is reduced in OA osteoblasts. In addition, the p42/44 mitogen-activated protein kinase (MAPK) pathway is higher in OA versus normal osteoblasts. This pathway can be triggered via IRS-1/Syp (the protein tyrosine phosphatase, non-receptor type 11) or growth factor receptor bound protein 2 (Grb2)/Src-homology collagen (Shc) interactions. Syp is highly phosphorylated in OA osteoblasts and its interaction with IRS-1 is very strong initially, yet rapidly dropped in the presence of IGF-I. The interaction of Grb2 with IRS-1 progressively increases in response to IGF-I in OA osteoblasts whereas this is absent in normal osteoblasts. This dysregulation is responsible for the fact that OA osteoblasts are more prone to proliferation than normal ones (Massicotte et al. 2006). Another important signaling pathway affected by OA is the Wnt (wingless-type MMTV integration site family member) signaling route and particularly the canonical Wnt/β-catenin signaling that is reduced. When correcting DKK2 levels in OA osteoblasts either directly by small interfering RNA (siRNA) techniques or indirectly by ablating the TGF-β amount, Wnt/β-catenin signaling activity is recovered (Chan et al. 2011). Conversely, when adding R-Spo2 to osteoblast cultures as a means to counteract their own weak production, Wnt/β-catenin signaling activity is also recovered (Abed et al. 2011). Like DDK2, R-Spo2 is under the control of TGF-β. Wnt/β-catenin signaling activity is also decreased by overproduction of SOST that is controlled by a low sirtuin 1 activity in OA osteoblasts (Abed et al. 2014). Phenotypic disturbance of OA osteoblast regarding Wnt signaling pathway is summarized in Fig. 2. TGF-β is a key player in the process and is regulated by HGF and leptin, themselves under the regulation of hypoxia. This latter can also directly induce TGF-β production.

In summary, the OA osteoblastic phenotype in cortical subchondral bone is more marked than in the trabecular one. Parathyroid hormone (PTH), IGF-I, and Wnt signaling and may be other pathways are disturbed. The right interaction between osteoblasts and the modified OA extracellular cartilage matrix (ECM) is disturbed too. TGF-β could be a key player in the process of OA in subchondral bone and hypoxia an aggravating factor of risk.

## Cell-matrix interface in OA (focus on the articular cartilage)

**Fig. 3:**
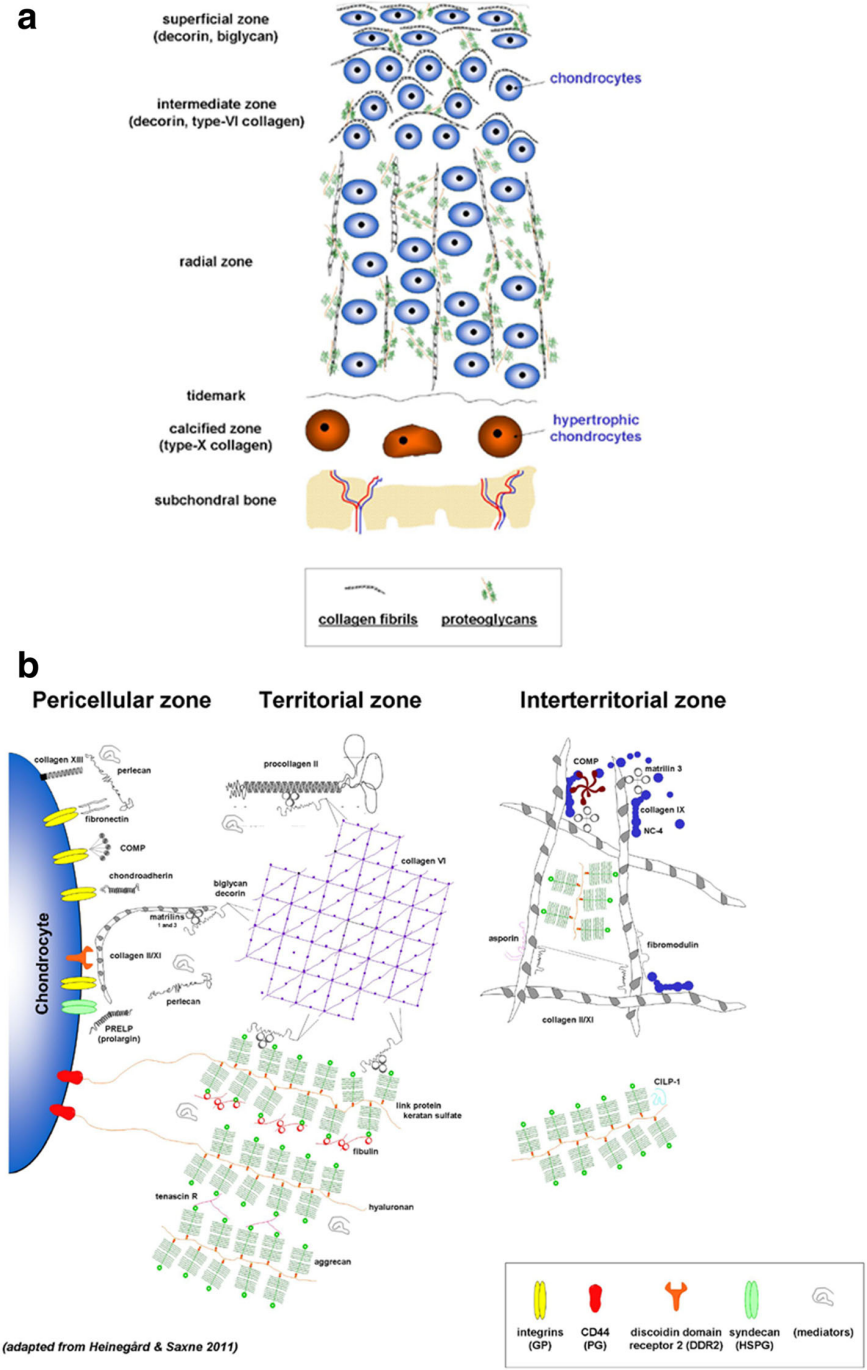
Organization of the normal adult articular cartilage. **a** Organization of the chondrocytes in their ECM in superficial, middle, and deep zones. The cement line separates the calcified zone from the subchondral bone. Major ECM components are presented in the box. **b** Normal PCM and cell-matrix interface (adapted from Heinegård & Saxne 2011)

**Fig. 4:**
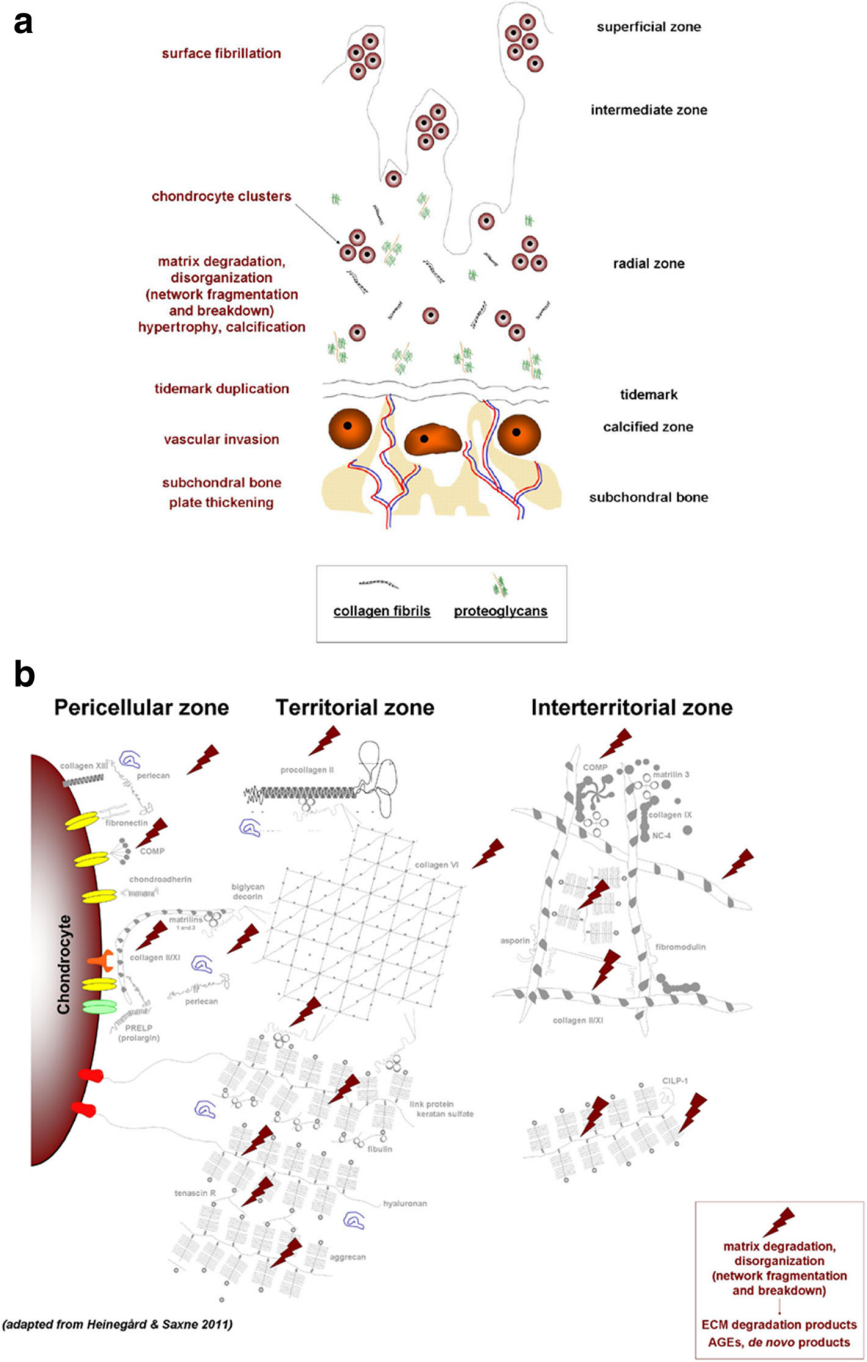
Pathological organization of OA cartilage. **a** Disrupted organization. **b** OA PCM and altered cell-matrix interface (adapted from Heinegård & Saxne 2011)

Cartilage degeneration is a hallmark of OA (Malemud et al. 1987). The articular chondrocytes are the only cell population present in the adult hyaline cartilage, the avascular, aneural tissue responsible for a smooth gliding of articulating surfaces. These cells are embedded within their ECM (mostly type-II collagen for tensile strength and proteoglycans with water for stiffness in compression/resilience) in three major, differently organized zones (Fig. 3a): a superficial (tangential) zone (cells parallel to the surface), a middle zone (spherical cells randomly organized), and a deep zone (columnar cells perpendicular to the surface). A calcified zone follows (small cells and type-X collagen fibrils), separated from the subchondral bone (subchondral bone plate and subarticular spongiosa) by the cement line. A narrow matrix surrounds the chondrocyte, namely the pericellular matrix (PCM) that is rich in perlecan, aggrecan monomers and small aggregates, hyaluronan, biglycan, collagens (type-VI and -IX collagen compared with the ECM), and fibronectin, followed by the territorial and interterritorial zones (Heinegård 2009, Poole et al. 1988, Wilusz et al. 2014) (Fig. 3b). In OA, as a result of various factors (pathological loading, obesity, aging, joint instability, repetitive stress injury, inflammation, genetic background), the cartilage becomes fibrillated in the superficial zone (loss of proteoglycans, reduction in water content, decreased elasticity) while cracks extend through the middle zone (formation of vertical matrix clefts, erosion of the collagen fibers), leading to cartilage breakdown towards the subchondral bone that becomes exposed and may be also deformed (Fig. 4a), with critical degradative changes also noted in constituents of the PCM (Heinegård & Saxne 2011) (Fig. 4b) and in association with synovial inflammation characterized by the production and release of inflammatory mediators and catabolic agents (cytokines: interleukin 1 beta - IL-1β and tumor necrosis factor alpha - TNF-α; chemokines: IL-8; proteinases: matrix metalloproteinases - MMPs - and aggrecanases; other mediators: PGE_2_, cyclooxygenase 2 - COX2, nitric oxide - NO, advanced glycation end products - AGEs) (Goldring & Otero 2011).

**Fig. 5:**
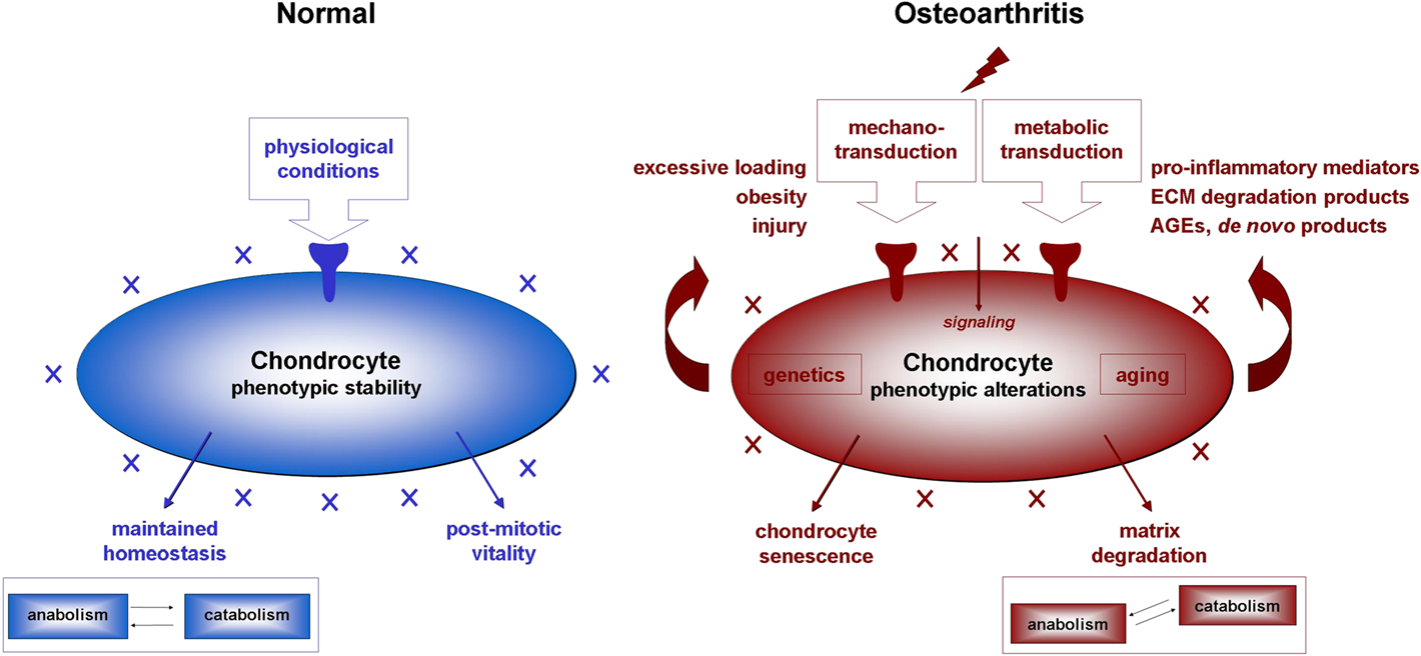
Cartilage homeostasis. Natural and pathological OA cellular and metabolic balance

**Fig. 6:**
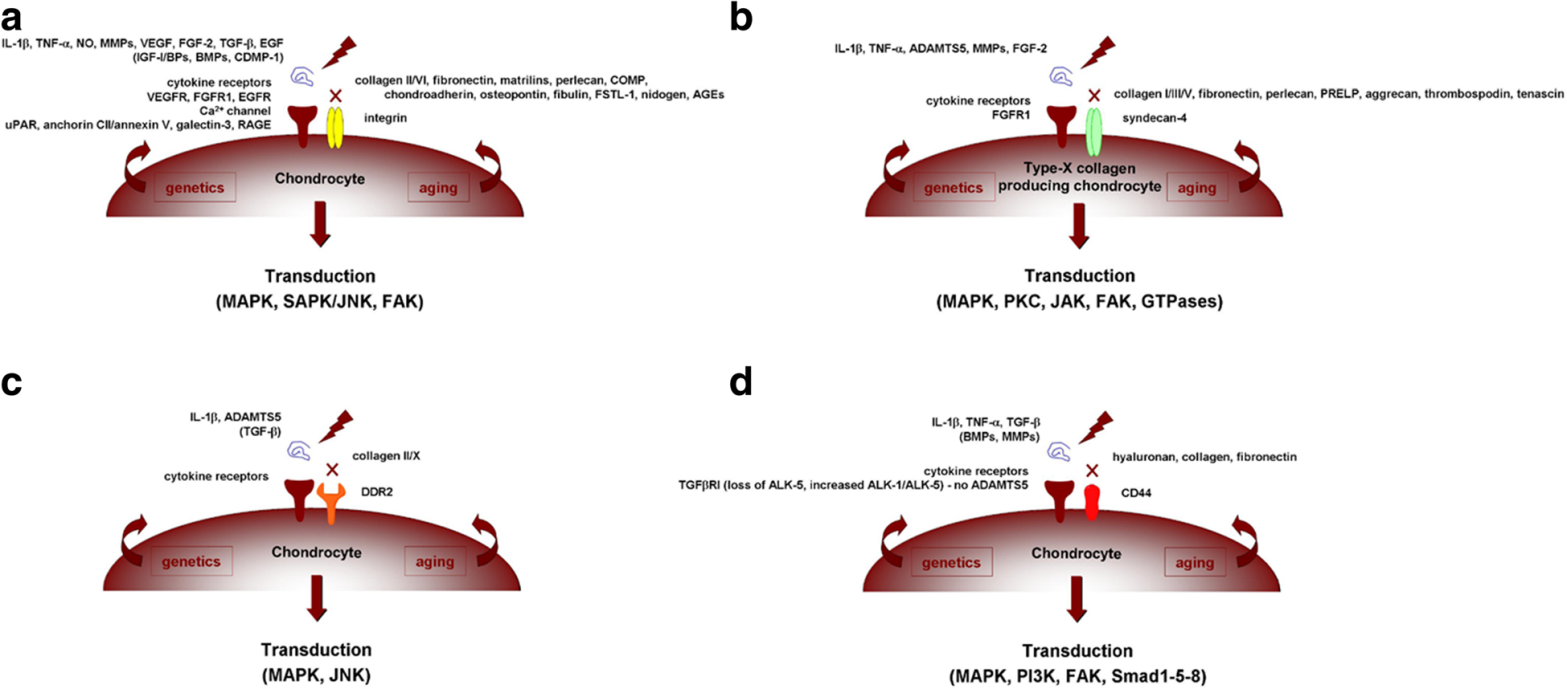
Cell-matrix interface in OA. Interactions between membrane sensors/receptors at the surface of chondrocytes become altered during OA, leading to abnormal signalling and activation of OA-related pathways via integrins (**a**), syndecan-4 (**b**), DDR-2 (**c**), and CD44 (**d**)

The chondrocytes are key cells implicated in the maintenance of the cartilage homeostasis by tightly controlling the production and degradation of ECM components according to the environment conditions in the joint (Goldring & Goldring 2007). They are capable of sensing biochemical and biomechanical stimulation to adapt the metabolic balance via interactions with PCM components that afford protection to the cells during loading (Goldring & Goldring 2007, Goldring & Otero 2011, Guilak et al. 2006). In normal adult cartilage, the chondrocytes are resting cells with a phenotypic stability characterized by a post-mitotic vitality with low metabolic activities (moderate matrix turnover) (Fig. 5), responding to physiological biochemical and biomechanical activation via membrane sensors that are also receptors for PCM molecules (Goldring & Goldring 2007, Guilak et al. 2006). Among them, the integrins are receptors for fibronectin and type-II and -VI collagen (Enomoto et al. 1993, Loeser 2014) acting with syndecan-4 as a co-receptor (Echtermeyer et al. 2009), discoidin domain receptor 2 (DDR-2) for type-II and -X collagen (Labrador et al. 2001, Lam et al. 2007, Leitinger & Kwan 2006), and CD44 for hyaluronan (Knudson 1993) (Fig. 6). Other mechanosensors include primary cilia, connexin 43, Indian hedgehog (Ihh), and the transient receptor potential vanilloid 4 (TRPV4) for collagens and proteoglycans (Knight et al. 2009, McGlashan et al. 2006, Muhammad et al. 2012, O’Conor et al. 2016, Ruhlen & Marberry 2014, Shao et al. 2012). In OA, the cells undergo phenotypic modifications reproducing those occurring during skeletal development (activation of cell proliferation processes, cell clustering, disruption of the metabolic balance with both abnormal matrix production and degradation, hypertrophy, and calcification) (Fig. 5) (Goldring & Goldring 2007, Loeser et al. 2012). Altered interactions between chondrocytes and PCM constituents are critical components of the pathogenesis of OA. As a result of cartilage matrix fragmentation, interactions between membrane sensors/receptors and PCM molecules become altered, leading to abnormal cell signalling that promotes the activation of matrix-degrading enzymes (MMPs, a disintegrin metalloproteinase with thrombospondin motifs - ADAMTS) and inflammatory cytokines or chemokines (IL-1β, TNF-α, NO, AGEs) and to the pathological release of mediators otherwise interacting with natural PCM elements that serve as a reservoir for such agents (vascular endothelial growth factor - VEGF, TGF-β, bone morphogenetic proteins - BMPs, basic fibroblast growth factor - FGF-2, IGF-I, epidermal growth factor - EGF, cartilage-derived morphogenetic protein 1 - CDMP-1) that may further stimulate OA-associated cascades (cell differentiation, hypertrophy, cytoskeletal reorganization, cell senescence, complement activation, altered autophagy…) (Arner & Tortorella 1995, Forsyth et al. 2002, Pulai et al. 2005, Stanton et al. 2002, Xu et al. 2007) (Fig. 6). Similar pathological changes and effects may also affect other cells relevant of the pathogenesis of OA, including synovial fibroblasts and bone cells as all joint tissues undergo phenotypic modifications in this disorder (Loeser et al. 2012), reflecting the high level of complexity and key involvement of cell-matrix interactions in human OA.

## Animal models of OA

The growing interest in both the factors triggering the development of OA and the therapeutic targets enabling more effective treatments has led to the introduction of a large number of animal models, depending on the many different aspects of human pathology. Each model can indeed describe only few peculiar aspects of the pathology and should be chosen accurately so as to maximize the outcome and the translatability of each study.

Primary OA is better monitored using naturally occurring models such as spontaneous and overuse-induced OA or genetically-induced OA whereas secondary OA can be mimicked by invasive surgical techniques such as meniscectomy, anterior cruciate ligament transaction (ACLT), or by chemical induction with inflammatory mediators or degenerative agents. Animal size and features are also crucial parameters for the study design. Although small animals allow for an easier and less costly management, their restricted size and differences with the human anatomy, histology, and physiology actually represent a limit to the translatability and investigation with radiologic techniques. On the other hand, large animals offer the advantages of spontaneous or readily inducible OA and of closer similarity to the human organism but at the same time, the high costs and negative public perception represent relevant drawbacks (Gregory et al. 2012).

During a study design, it is also important to adopt the model that better represents the pathology of interest so that the most suitable analyses can be performed. In OA, the most examined joint is the knee, however some models have been developed in metacarpophalangeal (McCoy 2015) and middle carpal joints (McIlwraith et al. 2010) of horses. When treating primary OA, naturally occurring models seem better suited since the disease spontaneously arises and simulates the natural progression of human pathology. In this model, one advantage is represented by the similarity between the experimental lesions and those occurring in human patients. Several animals have been proposed including mice, guinea pigs, rabbits, dogs, sheep, goats, and horses (Kuyinu et al. 2016, McCoy 2015). Guinea pigs represent a particularly good model to study human primary OA because of its rapid onset development (Yan et al. 2014). Horses are instead the most similar model to human individuals in terms of structure and pathology features and thus represent a valid system to study bone remodeling. Nevertheless, two major drawbacks of these models are represented by the long time needed for injury development and their high costs. Another way to obtain reliable models of primary OA is to use genetically-modified animals. This approach is useful to investigate the molecular basis of OA such as the effect of pro-inflammatory cytokines on its development (Little & Hunter 2013). Still, the complexity of this multifactorial pathology reduces the translatability to clinical practice of data derived from genetically-modified animals because therapeutic interventions targeting these specific genes do not take into account other contributing genes that participate in the pathogenesis of the disease (Miller et al. 2013).

Secondary OA commonly develops as a consequence of a traumatic event (post-traumatic OA) or as a result of other causes such as congenital conditions or bone disorders (Brown et al. 2006). This type of OA can be modeled by invasive (surgically- or chemically-induced OA) or non-invasive techniques (Table 2). Surgical models are usually adopted to study the drug effect on the disease process (Lampropoulou-Adamidou et al. 2014). These approaches ensure a rapid development of the pathology whereas their lack of correlation to natural degenerative changes in human OA represent a clear disadvantage. A typical surgical model is ACLT that causes joint destabilization leading to articular cartilage degeneration. The preferred animals adopted in this kind of model are the sheep and goat. Compared with meniscectomy, the OA lesions occurring in ACLT develop more slowly, making this approach more suitable for use in pharmacological studies (Proffen et al. 2012). A total or partial meniscectomy produces a great destabilization of the joint, thus leading to a rapid degeneration and a more severe pathology (McDermott & Amis 2006). A common model of OA in the mouse is the surgical destabilization of the medial meniscus (DMM) which leads to mild to moderate OA lesions primarily located in the anterior-central portion of the medial joint, with lesions on both the tibial plateau and femoral condyles (Glasson et al. 2007). Two other surgical models are represented by the medial meniscal tear model (transection of medial collateral ligament) which causes a proteoglycan and chondrocyte loss leading to cartilage degeneration and is generally performed in guinea pigs and rats (Glasson et al. 2007, McCoy 2015) and ovarectomy which is particularly indicated to investigate post-menopausal changes such as osteoporosis, eventually leading to OA (Martín-Millán & Castañeda 2013).

**Table 2:** Invasive and non-invasive models

	Invasive models	Non-invasive models
Advantages	rapid induction, high reproducibility, readily available, widely described systems	rapid induction, high reproducibility, low infection risk, long time frame of OA changes, allowing for early evaluation and intervention
Limitations	risk of infection, expertise needed, early changes often, undetectable because of rapid induction	not readily available, expertise needed, scarce literature

Another invasive way to induce OA is represented by the use of chemical compounds. It consists of the direct injection of toxic or inflammatory compounds into the joint such us papain, collagenase, sodium monoiodoacetate, and quinolones (bacterial arthritis) (Kuyinu et al. 2016).

Among the non-invasive models, the use of devices causing injury through controlled compressive loading and mechanical impact is the most common approach to produce over injury to a joint structure and promote OA. Such systems produce an external insult to the joint without the need of any chemical or surgical intervention, avoiding the risks of infection or of uncontrolled inflammation and allowing the creation of standardized injuries. Examples of non-invasive models are intra-articular tibial plateau fractures that reproduce post-traumatic OA from high energy impact and cyclic articular cartilage tibial compression mimicking chronic joint overuse (Christiansen et al. 2015). Application of adjustable mechanical loads and cyclic compression represent other alternatives to modify the cartilage structure and contribute to OA (Ko et al. 2013, Poulet et al. 2011). The choice of the right procedure to induce OA, as well as of the most adapted animal model depends on many factors such as the technical feasibility and the type of the pathology of interest since it could involve different development, progression, response to treatments, housing time and costs, animal amenability, and preferred outcome measurements (Table 3).

**Table 3:** Animal models

Animal	Size	Advantages	Limitations	Applications
Mouse	very small	low cost, amenability, standardized variants	post-operative management, low translability	chemically- and genetically-induced OA
Guinea pig	very small	low costs, similarity with human OA	sedentary behaviour, low translability	spontaneous and chemically-induced OA
Rat	small	low cost, amenability	post-operative management	surgically- and chemically-induced OA
Rabbit	small	amenability	self-regeneration, differences with human anatomy, post-operative management	surgically- and chemically-induced OA
Dog	medium/large	radiologic evaluation	high costs, perception	spontaneous and surgically-induced OA
Sheep/goat	large	amenability, radiologic evaluation, large joints	high costs, physiology	meniscectomy
Horse	large	radiologic evaluation	high costs, anatomy, perception	spontaneous OA and overuse

Another important aspect to take into account during the study design is the outcome of measurements. Quantitative methods comprise the evaluation of biomarkers, precursors, and products of metabolism released in the serum, urine, and synovial fluid whose levels can correlate with OA changes occurring in the joint. Histopathology and imaging like X-rays and MRI are suitable qualitative methods (Eckstein & Le Graverand 2015) that can be adopted together with appropriate histopathological scores such as the Mankin, O’Driscoll, International Cartilage Repair Society (ICRS), and Osteoarthritis Research Society International (OARSI) scores in order to provide a quantitative interpretation of the results.

In conclusion, translational animal research plays critical roles in helping researchers to understand the mechanisms of the disease, in improving the methods of early detection, and in identifying and investigating potential treatment targets for such a common, complex pathology as OA. From this point of view, it is crucial to identify and apply the most suitable model to each single research purpose in order to maximize translatability and comply with ethical an economical requirements. However, despite the potentiality of these models, the results arising out of preclinical studies have shown poor translation into clinical trials. One of the reasons could be that most studies involve post-traumatic OA, but the generated therapeutic intervention is then used to treat primary OA. Clearly defined post-traumatic OA in humans based on evaluations by MRI and other sensitive imaging approaches may represent a useful, more adapted clinical cohort for proof-of-principle for human trials. Moreover, a non-homogeneous data collection in these studies (animal husbandry conditions, animal gender…) could generate confusion and reduce the reliability of a study comparison. To improve uniformity and make the results more reproducible, it is strongly suggested to follow the Animal Research Reporting of In Vivo Experiments (ARRIVE) guidelines as well as to include skilled researchers, such as veterinary technicians and doctors in the research team.

### High tibial osteotomy models

Preclinical large animal models of high tibial osteotomy (HTO) can be used to test different types of osteotomies and to evaluate the effect of lower limb alignment on the reconstructive therapy of cartilage lesions and on the development and progression of OA (Allen et al. 1998, Lobenhoffer & Agneskirchner 2003).

**Fig. 7:**
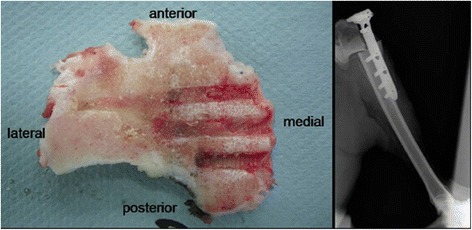
Late sequela of a monocortical screw fixation. Left: caudal view on a cross-sectional area of the proximal tibial segment showing monocortical screw imprints of the tibial head. Right: Post-operative X-ray of the right sheep tibia shortly after an open wedge HTO in a foundering animal with a secondary dislocation of the tibial head (from Pape & Madry, 2013)

Although many of our experimental knowledge on osteotomies is based on cadaver studies (Lim et al. 2011), the sheep as a preclinical, large animal model of HTO has not been described. Our goal was thus to establish such an HTO model as a means to study the effect of axial alignment on the lower extremity on specific issues of the knee joint such as in cartilage repair, development of OA, and meniscal lesions. Thirty-five healthy, skeletally mature, female Merino sheep underwent an HTO of their right tibia in a medial open-wedge technique inducing a normal (group 1, *n* = 12) and excessive valgus alignment (group 2, *n* = 12) and a closed-wedge technique (group 3, *n* = 11). The aim of surgery was to elucidate the effect of limb alignment on cartilage repair in vivo. The surgical technique as well as the clinical results have been published previously (Pape & Madry 2013). Severe post-operative complications occurred within 2–21 days after surgery. Two sheep were euthanized due to instability of the osteotomy that arose as a consequence of performing a monocortical fixation of the proximal screws (Fig. 7). Another sheep was euthanized after 4 days because of chronic patella dislocation caused by excessive valgus osteotomy and subsequently insufficient closure after medial arthrotomy (*n* = 1). Three intra-articular infections were noted 2–4 days after surgery, one infection in each osteotomy group. Although immediate revision with debridement was performed, all animals had to be euthanized 3–5 days after the surgical revision due to a progression of the systemic infection.

**Fig. 8:**
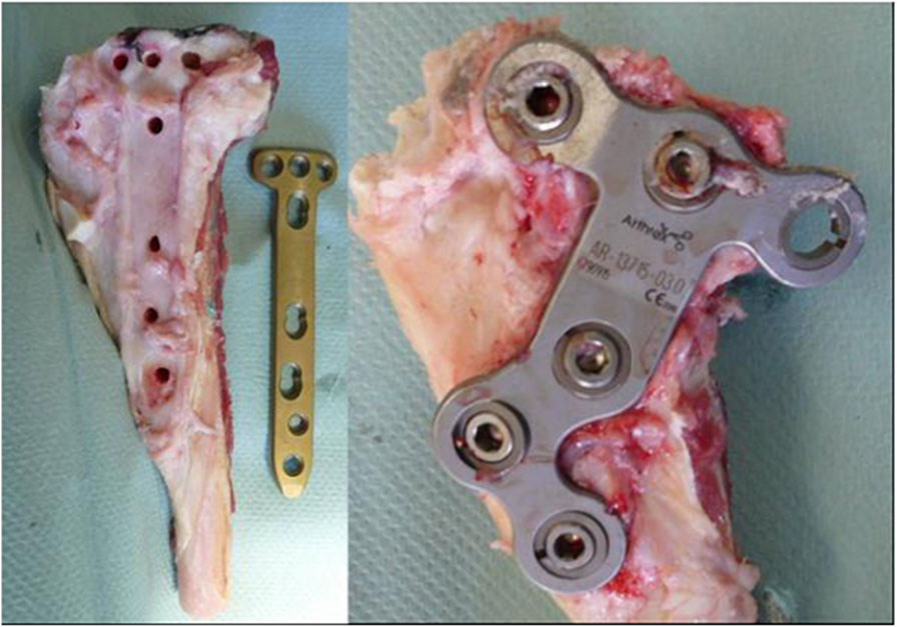
Plate design is crucial to meet the anatomical requirements of the sheep tibia. Proximally narrow, long and rigid plates (TomoFix small stature plate, Synthes, left) are advisable as opposed to anatomically shaped and broad plate designs (ContourLock, Arthrex, right) which fail to cover the sheep anatomy and may produce soft tissue impingement (from Pape & Madry, 2013)

In summary, solid bone healing and maintenance of correction are most likely to occur using the following surgical principles:

- . medial and longitudinal approach to the proximal tibia,
- . biplanar osteotomy to increase initial rotatory stability regardless of the direction of correction (Pape et al. 2013),
- . small, narrow but long implant with locking screws (small stature HTO plate, Synthes, Umkirch, Germany) (Fig. 8),
- . posterior plate placement to avoid slope changes, and
- . use of bicortical screws to account for the brittle bone of the tibial head and to avoid tibial head displacement (Tables 4 and 5).

**Table 4:** Surgical anatomical parameters relevant for HTO in humans and sheep

	Human knee	Sheep stifle joint	Surgical consequences for the sheep HTO model
Tibial plateau width [mm]	60-70	46-56	match screw length, narrow and strait plate design necessary
Tibia valga [°]	0	3.5	valgus overcorrection more likely
Knee range of motion [°]	0-0-140	0-35-72	dorsal plate positioning after open wedge HTO suggested due to increased loading of the posterior tibial plateau
Tibial tuberosity dimension adding to the anterio-posterior diameter of the tibial head [%]	10-15	30-35	anterior plate misplacement more likely
Tibial tuberosity height distance in relation to the joint line [mm]	25-30	10-15	anterior plate misplacement more likely
Posterior slope of the posterior articular surface [°]	0-10	20 ± 3	narrow and straight plate design necessary for posterior placement
Biomechanical properties of the tibial head	elastic cortical bone, exuberant amount of spongious bone	brittle cortical bone, few spongious bone	bicortical proximal screw placement mandatory to avoid fracture and dislocation, biplanar osteotomy mandatory regardless of the desired direction of correction
Musculature of the hind limb	remote from bony knee structures	voluminous on medial and lateral side of the femur	distal femoral and proximal lateral tibial osteotomy almost impossible, stay on the medial side of the proximal tibia for any desired correction angle
Trochlea ridge	lateral ridge extending further laterally and anteriorly	medial ridge extending further cranially and dorsally than lateral ridge	higher propensity of patella instability after valgus correction

Case reports: + (seldom), ++ (infrequent), +++ (frequent), 1 (may depend on implant design)

° degree

**Table 5:** Probabilities of potential pitfalls among humans and sheep

Structure	Human	Sheep
Neurologic injuries	++	-
Osteonecrosis of proximal fragment	++	-
Fracture through proximal fragment with violation of the joint space	++	-
Infection	+	+++
Vessel injury	+	++
Bone cysts without screw perforation	-	+
Compartment syndrome	+	-
Non-union	+	-
Loss of correction due to implant failure	1	+
Overcorrection	1	-

Case reports: + (seldom), ++ (infrequent), +++ (frequent), 1 (may depend on implant design)

Although successful HTO in sheep is complex, this model may serve as an elegant model to induce axial malalignment in a clinically relevant environment and osteotomy healing under challenging mechanical conditions due to its similarities with humans.

## Stem cells in OA

Mesenchymal stem cells (MSCs) are considered a promising option for the treatment of OA. MSCs are multipotent progenitor cells with capability of self-renewal, high plasticity, immunosuppressive and anti-inflammatory action, and the possibility to differentiate into selected lineages including chondrocytes (Manferdini et al. 2013). Moreover, these cells, derived from perivascular cells called "pericytes", play a key role in the response to tissue injury not only by differentiating themselves but also by inducing repair/regeneration processes at the site of damage through the secretion of several bioactive molecules (Caplan & Correa 2011).

However, despite extensive preclinical research and promising findings reported in the clinical practice upon surgical application or via minimally invasive treatments through intra-articular injections (Filardo et al. 2013), both their potential and limitations for a thorough use in patients remain controversial (de Girolamo et al. 2016). Different cell sources have been explored as solutions for cartilage lesions and OA treatment such as bone marrow, adipose tissue, synovial tissue, and peripheral blood. Altogether, a recent systematic review identified 60 studies of which only seven were randomized (Filardo et al. 2016) (Table 6). On the other hand, the available studies allow to draw some indications. First, no major adverse events have been reported. Second, a clinical benefit has been shown in most of the studies regardless of the cell-source processing method and clinical indication, with clinical improvements and also positive MRI and macroscopic findings. Moreover, some factors that may influence the outcome have been identified, such as age, body mass index (BMI), lesion size or degenerative stage. Beside the many still open questions in terms of the choice of cell source, the best processing method, the dose and administration method as well as the best treatment indications, the overall results also underlined only a partial benefit provided by this biological approach to treat early OA stages, both in terms of uncomplete recovery and compared with the benefit obtained with other available treatment options.

**Table 6:** Stem cells for OA treatment: clinical evidence from randomized controlled trials

Treatment	Procedure	Patients	Follow-up	Outcomes	References
BMSCs	i.a.	15 (versus HA)	1 year	improved function, higher cartilage quality (MRI)	Vega et al. 2015
BMSCs (HA)	i.a. (meniscectomy)	18 (low versus high MSCs dose versus HA)	2 years	pain improvement, meniscus regeneration (MRI)	Vangsness et al. 2014
BMSCs (HA)	i.a. (osteotomy and microfracture)	28 (versus HA)	2 years	clinical improvements, higher cartilage quality (MRI)	Wong et al. 2013
BMCs (matrix/PEMF)	surgical delivery (collagen matrix)	15 (versus matrix)	1 year	improved recovery	Cadossi et al. 2014
PBSCs (HA)	i.a. (drilling)	25 (versus HA)	2 years	improved cartilage quality (MRI)	Saw et al. 2013
ASVF	surgical delivery (microfracture)	40 (versus microfracture)	2 years	clinical improvements, higher cartilage quality (MRI)	Koh et al. 2016
ASVF (PRP)	i.a. (osteotomy)	21 (versus PRP)	2 years	clinical improvements, fibrocartilage coverage	Koh et al. 2014

*Abbreviations*: *MSCs* mesenchymal stem cells, *BMSCs* bone marrow-derived MSCs, *HA* hyaluronic acid, *BMCs* bone marrow concentrates, *PEMF* pulsed electromagnetic field, *PBSCs* peripheral blood stem cells, *ASVF* adipose stromal vascular fraction, *PRP* platelet-rich plasma, *i.a*. intra-articular

Basic science results may be useful to understand the suboptimal results obtained clinically so far and to help identifying development strategies for future directions to improve OA treatments. The safety of using MSCs is supported by preclinical studies but some risks have been also underlined such as the possibility of contamination during cell manipulation and the formation of free body and scar tissue in the joint with the transplantation of large cell numbers (Agung et al. 2006). The risk of unwanted differentiation and inadequate aging is another possibility strictly related to the treatment efficacy itself. In fact, signs of hypertrophy have been documented with MSC chondrogenic differentiation: type-X collagen, MMP13, alkaline phosphatase (ALP) are signs of terminal differentiation that may lead to mineralization in vivo and thus to the formation of a low quality cartilage (Hellingman et al. 2012). These findings have been documented not only in bone marrow-derived cells but also when dealing with adipose tissue and even with embryonic stem cells, suggesting that the current culture conditions may be not ideal to guide MSCs toward chondrogenic differentiation. Different strategies have been proposed to inhibit terminal differentiation but nowadays no hyaline cartilage regeneration can be obtained in vitro, yet, and therefore no hyaline cartilage may be expected in vivo (Hellingman et al. 2012). Other indications from the preclinical literature relate to the number of cells and the need for either expansion or concentration. Heterogeneous findings do not allow to drag clear conclusions on this matter but the need to obtain many cells through expansion has been questioned by studies showing similar or even better results through the use of concentrates which may offer a lower number of cells but on the other hand the benefit of administering MSCs in their niche and therefore allowing them to act in more favorable conditions (Perdisa et al. 2015, Wu et al. 2016). Regardless of the cell type and method of manipulation, MSCs are rarely applied in clinical practice alone and platelet concentrates have been frequently associated to their use. Even though no randomized controlled trials has been performed to demonstrate the added benefits of blood-derived products, basic science supports platelet-rich plasma (PRP) combination, showing a synergistic effect through cell-proliferation and extracellular synthesis, anti-inflammatory effects, and in the end lower OA progression (Yun et al. 2016).

In conclusion, the state of the art of MSCs for the treatment of OA shows contrasting findings. While promising results have been obtained in terms of clinical improvement, results are not optimal and there is still a lack of indication on the optimal cell source, processing, and administration modality. Further studies are needed both preclinically to develop appropriate models to further explore the potential of MSCs as well as in the clinical practice through randomized controlled trials to clearly demonstrate the advantages of this biological approach with respect to the other available treatments for OA.

## Tissue engineering in OA

The ideal treatment for OA should control not only the symptoms but also slow down or prevent the degradation of the joint. In this sense, tissue engineering may be a game changer in the treatment of OA as it can possibly create functional substitutes through integrated solutions where scaffolds, cells and drugs/growth factors are used, alone or in combination (Langer and Vacanti 1993).

Cell-free implants consisting of matrices and/or local factor delivery systems for progenitor cell recruitment and differentiation have been investigated. The process can promote the recruitment of MSCs from the bone marrow or synovium into a defect-filling chondroinductive matrix where they form new cartilage. Microfracture can be also applied to create small channels between the defect and subchondral bone to improve the contact between the defect and bone marrow (Ringe et al. 2012). In situ stem cells administration by intra-articular injection can result in the engraftment of cells on the meniscus, fat pad, and synovium with regeneration of meniscal tissue and protection of the cartilage, but no engraftment has been seen on the fibrillated cartilage surface (Murphy et al. 2003). Vectors have been used for ex vivo intra-articular gene delivery. It has been reported that hydrated collagen-glycosaminoglycan matrices containing adenoviral vectors promote localized reporter gene expression in vivo for at least 21 days after implantation into osteochondral defects localized in rabbit knees (Pascher et al. 2004). Interestingly, the delivery of autologous chondrocytes or of MSCs for cartilage regeneration may be promising when envisioning to treat early OA upon hydrogel administration (Vilela et al. 2015). This can be performed directly after debridment in the knee articular cavity or at damaged sites in order to improve mechanical stability of cartilage while facilitating tissue regeneration. The advantages of hydrogel systems are as follows:

- . they are made of a three-dimensional (3D) permeable polymer network,
- . they are produced by a reaction of monomers or by hydrogen bounding,
- . they exhibit a hydrophilic structure making them capable of holding large amounts of water (thousands of times their dry weight),
- . their mechanical properties (e.g. stiffness) can be tuned according to the application,
- . they have free-shapeable properties (bending, knotting stretching, compressing),
- . they can be injected in minimally invasive manner,
- . they have an ability to encapsulate and control the delivery of cells and growth factors, and
- . they are bioadhesive and biocompatible.

Table 7 summarizes the relevant studies dealing with the application of hydrogels in early OA as cell/drug delivery carriers.

**Table 7:** Application of hydrogels as cell/drug delivery carriers for OA therapy

Hydrogels	Objectives	References
HA	injection of autologous MSCs in goat knee joints	(Murphy et al. 2003)
PlnD1-HA microgel	injection of PlnD1-HA to potentiate BMP-2 activity in an experimental murine OA model	(Srinivasan et al. 2012)
Alginate-chitosan beads	injection of beads to reduce OA in rabbits	(Oprenyeszk et al. 2013)
Kartogenin-conjugated chitosan hydrogel	intra-articular delivery to protect against OA in rats	(Kang et al. 2014)
Fibrin/HA-MA hydrogel	induction of bone marrow-derived MSCs chondrogenesis	(Snyder et al. 2014)
Gelatin hydrogels incorporating rapamycine micelles	intra-articular administration to protect against OA in mice	(Matsuzaki et al. 2014)
CM-SIN and GelMA hydrogels	intra-articular injection to reduce OA in mice	(Chen et al. 2016)

*Abbreviations*: *HA* hyaluronic acid, *PlnD1* perlecan module, *MA* methacrylic anhydride, *CM-SIN* sinomenium encapsulated in chitosan microspheres, *GelMA* gelatine methacrylate, *MSCs* mesenchymal stem cells, *BMP-2* bone morphogenetic protein 2, *OA*, osteoarthritis

Our group has proposed the use of gellan gum (GG) hydrogel, a linear anionic exopolysaccharide (molecular mass: ~ 10^6^ g/mol) that exists in two forms (high acyl - HA, low acyl - LA) for cartilage tissue engineering (Oliveira et al. 2010). GG hydrogels are promising systems for OA treatment strategies as:

- . they can be used as injectable systems for minimally invasive surgeries,
- . they allow for gelation in the presence of body fluids within few seconds,
- . they display some degree of bioadhesiveness while allowing cells encapsulation, proliferation, and differentiation towards the chondrogenic lineage, and
- . they are noncytotoxic.

The methacrylated GG derivative (GGMA) has been synthesized and processed in the form of hydrogel and scaffolds (single or multilayered) to deliver cells and drugs for cartilage regeneration (Pereira et al. 2014). These systems are now also being tested in preclinical assays to provide a valid alternative to hyaluronan in VS strategies and cell delivery for the treatment of early OA. GG-based hydrogel systems are valuable technological platform for the delivery of stem cells using minimally invasive techniques as it is an injectable system that mimics the native cartilage ECM.

Future tissue engineering approaches using hydrogels in OA should:

- . be patient-specific,
- . be based on a single step surgical procedure,
- . exploit the combination of different cell types (e.g. co-administration of stem cells and chondrocytes) alone or together with growth factors or PRP, and
- . use systems with tunable degradation rates for delivery of drugs and cells (drug concentration, cell density, passage, and cell source should be evaluated).

The patient selection is also an important criterion in future clinical trials to dictate the validity and efficacy of the tissue engineering regenerative strategies for safe translation in the daily clinical practice.

## Conclusions

Our current knowledge on OA as a severe human disorder for which there is no reliable cure to date has greatly increased from a basic point of view due to the availability of a variety of clinically relevant animal models, involving various, fine mechanisms that involve all the tissues in the joint. The identification of the processes and mediators underlying the initiation and progression of OA provides hope to establish adapted, novel clinical treatments with a strong emphasis on the use of MSCs and on tissue engineering approaches to deliver effective and safe therapeutic compounds in patients in a close future.
